# Retraction: Protective Effects of Essential Oils as Natural Antioxidants against Hepatotoxicity Induced by Cyclophosphamide in Mice

**DOI:** 10.1371/journal.pone.0344547

**Published:** 2026-03-10

**Authors:** The PLOS One Editors

Following the publication of this article [1] concerns were raised with results presented in Figs 3 and 4. Specifically,

- The following panels appear to partially or fully overlap, despite being used to represent different experimental conditions or samples obtained from different animal species, calling into question the reliability of the associated quantified results:
- All β-actin panels in Figs 2 and 3 of this article [1] and all β-actin panels in Figs 1 of [2], [3], and [4].
- The Fig 3 C-reductase panel, the Fig 3C CYP2C6 panel, and lanes 8–10 of the Fig 3 CYP3A4 panel.
- Similarities were noted between multiple regions within Fig 4C.

In light of these concerns, which call into question the integrity and reliability of the published results, the *PLOS One* Editors retract this article.

All authors either did not respond directly or could not be reached.
